# Micropapillary breast carcinoma in comparison with invasive duct carcinoma. Does it have an aggressive clinical presentation and an unfavorable prognosis?

**DOI:** 10.1186/s12885-024-12673-0

**Published:** 2024-08-12

**Authors:** Yasmine Hany Abdel Moamen Elzohery, Amira H. Radwan, Sherihan W. Y. Gareer, Mona M. Mamdouh, Inas Moaz, Abdelrahman Mohammad Khalifa, Osama Abdel Mohen, Mohamed Fathy Abdelfattah Abdelrahman Elithy, Mahmoud Hassaan

**Affiliations:** 1 Department of General Surgery, Faculty of Medicine, Ain Shams University, Cairo, Egypt; 2 Department of Radiodiagnosis, NCI, Cairo University, Giza, Egypt; 3 Department of Pathology, National Cancer Institute, Cairo University, Giza, Egypt; 4 Department of Epidemiology and Preventive Medicine, National Liver Institute, Menoufia, Egypt; 5 Baheya Center for Early Detection and Treatment of Breast Cancer, Giza, Egypt; 6 Present Address: Department of Surgical Oncology, Faculty of Medicine, Al Azhar University, Cairo, Egypt; 7 Present Address: Departement of Surgical Oncology, National Cancer Institute, Cairo University, Giza, Egypt

**Keywords:** IMPC, IDC, LVI

## Abstract

**Background:**

Invasive micropapillary carcinoma (IMPC) was first proposed as an entity by Fisher et al. In the 2003 World Health Organization (WHO) guidelines for histologic classification of the breast tumors. IMPC was recognized as a distinct, rare histological subtype of breast cancer.

IMPC is emerging as a surgical and oncological challenge due to its tendency to manifest as a palpable mass, larger in size and higher in grade than IDC with more rate of lymphovascular invasion (LVI) and lymph node (LN) involvement, which changes the surgical and adjuvant management plans to more aggressive, with comparative prognosis still being a point of ongoing debate.

**Aim of the study:**

In this study, we compared the clinicopathological characteristics, survival and surgical management of breast cancer patients having invasive micropapillary carcinoma pathological subtype in comparison to those having invasive duct carcinoma.

**Method:**

This is a comparative study on female patients presented to Baheya center for early detection and treatment of breast cancer, in the period from 2015 to 2022 diagnosed with breast cancer of IMPC subtype in one group compared with another group of invasive duct carcinoma. we analyzed 138 cases of IMPC and 500 cases of IDC.

**Results:**

The incidence of LVI in the IMPC group was 88.3% in comparison to 47.0% in the IDC group (p < 0.001). IMPC had a higher incidence of lymph node involvement than the IDC group (68.8% and 56% respectively). IMPC had a lower rate of breast conserving surgery (26% vs.37.8%) compared with IDC.

The survival analysis indicated that IMPC patients had no significant difference in overall survival compared with IDC patients and no differences were noted in locoregional recurrence rate and distant metastasis rate comparing IMPCs with IDCs.

**Conclusion:**

The results from our PSM analysis suggested that there was no statistically significant difference in prognosis between IMPC and IDC patients after matching them with similar clinical characteristics. However, IMPC was found to be more aggressive, had larger tumor size, greater lymph node metastasis rate and an advanced tumor stage.

## Introduction

Breast cancer is the most common cancer in women. In the 2012 World Health Organization (WHO) classification of breast cancer. Breast Cancer is classified into up to 21 different histological types depending on cell growth, morphology and architecture patterns [1]. The invasive carcinoma of no special type (IBC-NST), which is known as invasive ductal carcinoma (IDC), is the most frequently occurring histological type, which constitutes around 75% of invasive breast carcinoma [2].

Invasive micropapillary carcinoma (IMPC) was first proposed as an entity by Fisher et al. in 1980 [3] and first described as the term “invasive micropapillary carcinoma” by Siriaunkgul et al. [4] in 1993.

In the 2003 World Health Organization (WHO) guidelines for histologic classification of the breast tumors [5]. IMPC was recognized as a distinct, rare histological subtype of breast cancer. While micropapillary histological architecture is present in 2–8% of breast carcinomas, pure micropapillary carcinoma is uncommon and accounts for 0.9–2% of all breast cancers [6].

IMPC exhibits more distinct morphologic architecture than the IDC, characterized by pseudopapillary and tubuloalveolar arrangements of tumor cell clusters in clear empty sponge-like spaces that resemble extensive lymphatic invasion [7]. The neoplastic cell exhibits an “inside-out” pattern, known as the reverse polarity pattern [2].

Most studies demonstrate that the radiological findings of IMPC are irregular-shaped masses with an angular or spiculated margin on ultrasound, mammography and MRI with heterogeneous enhancement and washout kinetics on MRI [8].

IMPC had tendency to manifest as a palpable mass, larger in size and higher in grade than IDC with more rate of lymphovascular invasion (LVI) and lymph node (LN) involvement, which changes the surgical and adjuvant management plans to more aggressive, with comparative prognosis still being a point of ongoing debate [9].

### Aim of the study

In this study, we compared the clinicopathological characteristics, survival and surgical management of breast cancer patients having invasive micropapillary carcinoma pathological subtype in comparison to those having invasive ductal carcinoma.

### Patient and method

This is a comparative study on female patients presented to Baheya center for early detection and treatment of breast cancer, in the period from 2015 to 2022 diagnosed with breast cancer of IMPC subtype in one group compared with another group of invasive duct carcinoma.

This retrospective study analyzed 138 cases of IMPC and 500 cases of IDC. Informed consent was obtained from all patients. Ethical approval is obtained from Baheya center for early detection and treatment of breast cancer and National research center ethics committee. Baheya IRB protocol number:202305150022.

The following clinical-pathological features were analyzed for each case: patient age at diagnosis, clinical presentation, laterality, imaging findings, histopathological examination, treatment plan with either primary surgical intervention or other treatment protocol according to tumor stage and biological subtypes.

A breast pathologist evaluated the tumor size, type, grade, lymphovascular invasion, estrogen receptor (ER), progesterone receptor (PR), human epidermal growth factor receptor 2 (HER2) receptor and the axillary lymph node involvement.

According to the ASCO/CAP guideline update, 2019: Samples with 1% to 100% of tumor nuclei positive for ER or progesterone receptor (PgR) are interpreted as positive. If ER (not PgR), 1% to 10% of tumor cell nuclei are immunoreactive, the sample are reported as ER Low Positive. There are limited data on the overall benefit of endocrine therapies for patients with low level (1%-10%) ER expression, but they currently suggest possible benefit, so patients are considered eligible for endocrine treatment. A sample is considered negative for ER or PgR if < 1% or 0% of tumor cell nuclei are immunoreactive [10]. An Allred score between 0 and 8. This scoring system looks at what percentage of cells test positive for hormone receptors, along with how well the receptors show up after staining, called intensity: proportion of cells staining (0, no staining; 1, < 1%; 2, between 1 and 10%; 3, between 11 and 33%; 4, between 34 and 66% and 5, between 67%–100% of the cells staining). Intensity of positive tumor cells (0, none; 1, weak, 2, intermediate; and 3, strong) [11].

HER2 Test Guideline IHC Recommendations, 2018. IHC 0: as defined by no staining observed or membrane staining that is incomplete and is faint/barely perceptible and within <  = 10% of the invasive tumor cells. IHC 1 + : as defined by incomplete membrane staining that is faint/barely perceptible and within > 10% of the invasive tumor cells. IHC 2 + : The revised definition of IHC 2 + (equivocal) is weak to moderate complete membrane staining observed in > 10% of tumor cells. IHC 3 + : based on circumferential membrane staining that is complete, intense in > 10% of tumor cells. [12].

ASCO–CAP HER2 SISH Test Guideline Recommendations,2018 Twenty nuclei (each containing red (Chr17) and black (HER2) signals) should be enumerated. The final results for the HER2 status are reported based on the ratio formed by dividing the sum of HER2 signals for all 20 nuclei divided by the sum of Chromosome 17 signals for all 20 nuclei. The amplification status is defined as Amplified if the HER2/Chromosome 17 ratio > / = 2.0 and the average Her2 gene copy number is > / = 4.0. It is non-Amplified if the HER2/Chromosome 17 ratio < 2.0 with the Her2 gene copy number is < 4.0. If the HER2/Chr17 ratio is < 2 and the Her2 gene copy number is between 4.0 and 6.0, or, HER2/Chr17 ratio is > / = 2 and the Her2 gene copy number is < 4, or HER2/Chr17 ratio is < 2 and the Her2 gene copy number is > / = 6.0, an additional work should be done. [12].

Follow-up duration was calculated from the date of diagnosis to the date of the last follow-up. Patients still alive at the last follow-up censored or to the date of occurrence of any event or death.

Disease-free survival was defined as the duration (months) from the initial diagnosis of breast cancer to first any type of recurrence (invasive ipsilateral breast tumor recurrence, local invasive recurrence, regional invasive recurrence, invasive contra lateral breast cancer, distant metastasis.

Overall survival (OS) is defined as the time from diagnosis of breast cancer to death from any cause.

Data were statistically analyzed using an IBM-compatible personal computer with Statistical Package for the Social Sciences (SPSS) version 23. Quantitative data were expressed as mean, standard deviation (SD) and range (minimum–maximum). Qualitative data were expressed as Number (N) and percentage (%), while A P value of < 0.05 was statistically significant. For comparison of unmatched data, chi-square tests were used for categorical variables and t-tests or Mann–Whitney tests for continuous variables.

In this study, we analyzed 138 cases of IMPC which presented to our center in the period from 2015 to 2022.We included a total number of 500 cases of IDC as controls with a ratio of controls to cases 4:1.

Propensity score matching (PSM) is a method for filtrating experimental and control cases of similar characteristics, which are called the matching variables, from existing data to make them comparable in a retrospective analysis. PSM reduce the effect of selection bias. So, the comparison of outcomes between two groups can be fair.

The variables for propensity score matching were selected as follows: age (years), tumour size (cm), nodal status, HR status and HER2 status.

To diminish the effects of baseline differences and potential confounds in clinical characteristics and patients across histology subtypes for outcome differences (disease-free survival and overall survival), PSM method was applied with each micropapillary patient matched to one IDC patient who showed similar baseline characteristics in terms of: menopausal status, comorbidities, multiplicity, histologic grade, tumor size, stage, nodal status, ER /PR status. Differences in prognosis were assessed by Kaplan–Meier analysis.

## Result

Most of the patients were postmenopausal, the mean age of patients in IMPC group was 57.36 ± 11.321 years while the mean age of the IDC group was 56.63 ± 9.719 years (*p* = 0.45) (Table 1).

**Table 1:** Socio-demographic data of the studied groups

	IDC *N* = 500	Micropapillary *N* = 138	*p*-value
Age	56.63 ± 9.719	57.36 ± 11.321	0.45
Menopausal status Post-menopausal Pre-menopausal	334 66.8% 166 33.2%	115 83.3% 23 16.7%	< 0.001
Co morbidities No Yes (hyper tension, diabetes, ischemic heart disease and liver disease)	218 43.6% 282 56.4%	110 79.7% 28 20.3%	< 0.001

**Fig. 1:**
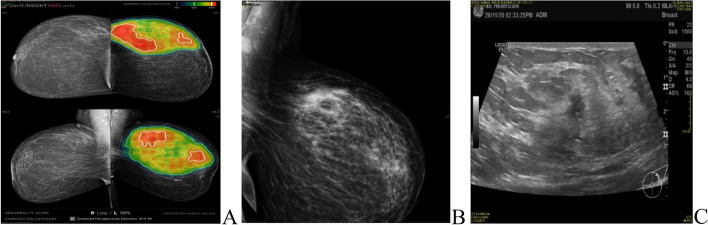
**A**, **B** 37-years-old female patient presented with Left breast UOQ extensive fine pleomorphic and amorphous calcifications of segmental distribution, with UOQ multiple indistinct irregular masses. **C** ultrasound showed left breast UOQ multiple irregular hypoechoic masses with calcific echogenic foci, the largest is seen at 1 o’clock measuring 13 × 15mm. Intraductal echogenic lesions are noted

**Fig. 2:**
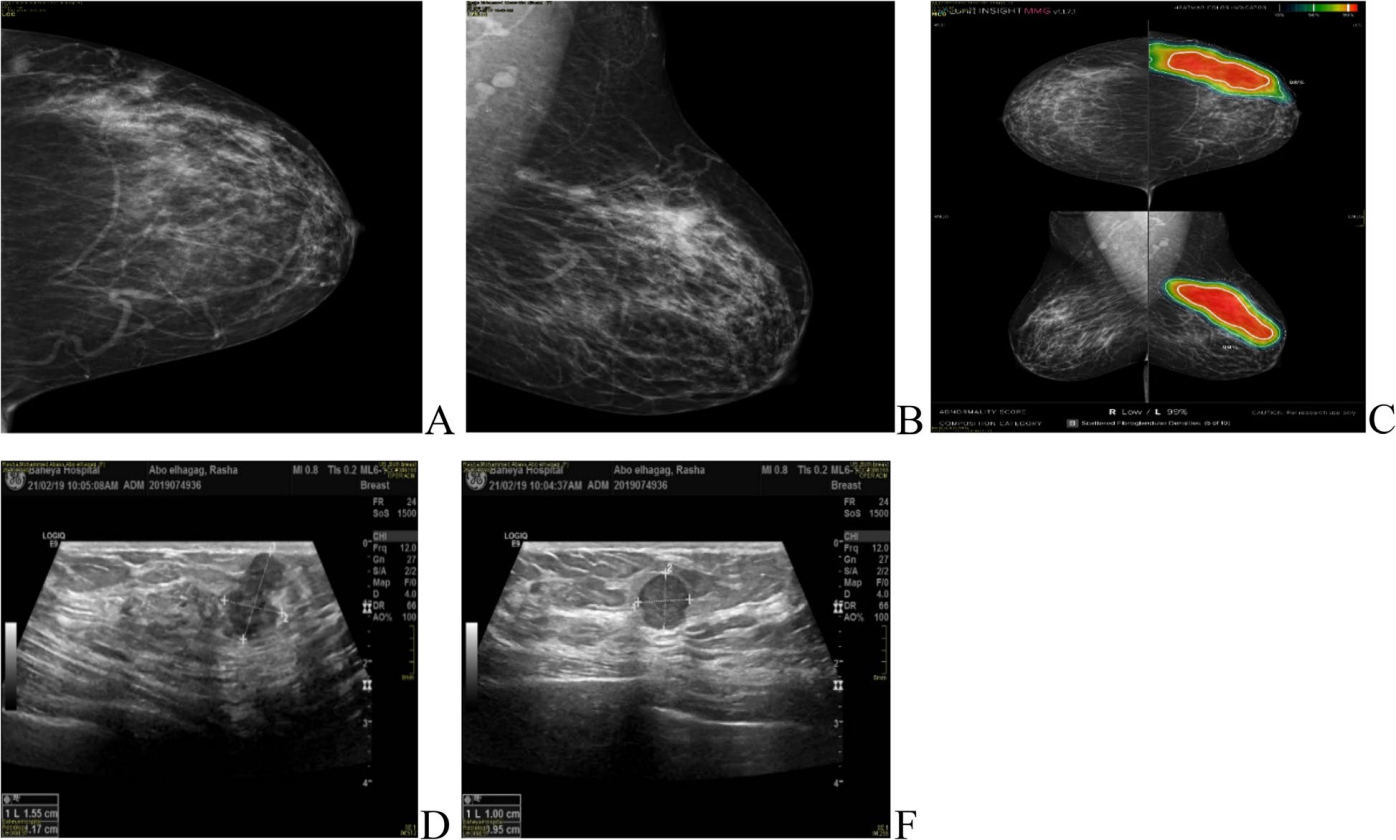
**A**, **B**, **C** 40-years-old female patient presented with left UOQ extensive pleomorphic microcalcifications of segmental distribution reaching the areola, with multiple well-circumscribed small obscured masses. **D**, **E** complementary Ultrasound showed left 2 o’clock multiple ill-defined and well-defined hypoechoic masses (BIRADS 5)

The most common presentation of IMPC on breast mammography was an irregular shaped mass with a non-circumscribed spiculated margin. while, the most common sonographic finding of IMPC was hypoechoic mass with irregular shapes and spiculated margins. Associated microcalcifications were found in 49 patients (35.5%) of IMPC group. Figs. (1, 2): Radiological characteristics of IMPC.

All patients underwent axillary sonography where 77 patients (55.8%) of the IMPC group exhibited pathological lymph nodes and 18 patients (13%) had indeterminate lymph nodes demonstrating preserved hila and associated with either a symmetrical increase of their cortical thickness reaching 3mm or with a focal increase in the cortical thickness.

Multiple lesions were detected in 30% of IMPC patients in comparison to 7% of IDC patients. Intra-ductal extension with nipple involvement was found in 44 patients (31.9%) of the IMPC group (Table 2).

**Table 2:** Imaging features of the studied groups

	IDC *N* = 500	Micropapillary *N* = 138	*p*-value
Side RT LT Bilateral	247 49.4% 250 50% 3 0.6%	66 47.8% 68 49.3% 4 2.9%	
Site LIQ LOQ Retroareolar UIQ UOQ	20 4.0% 27 5.4% 30 6.0% 50 10.0% 373 74.6%	10 7.2% 7 5.1% 3 2.2% 27 19.6% 91 65.9%	0.005
mass size /cm	2.72 ± 1.395	3.37 ± 1.81	< 0.001
Mammo T T1 T2 T3 T4	110 22.6% 244 48.8% 56 11.2% 87 17.4%	40 28.8% 73 52.8% 15 10.8% 10 7.2%	0.03
Mammo dense ACR A ACR B ACR C	22 4.4% 391 78.2% 87 17.4%	6 4.3% 107 77.5% 25 18.1%	0.98
Mammo (Single/MC/MF) Multicentric Multifocal Single	22 4.4% 14 2.8% 464 92.8%	23 16.7% 19 13.8% 96 69.6%	< 0.001
BIRADs 3 4a 4b 4c 5 6	3 0.6% 6 1.2% 18 3.6% 90 18.0% 344 68.8% 39 7.8%	1 0.7% 5 3.6% 6 4.3% 27 19.6% 86 62.3% 13 9.4%	0.41
Axillary lymph node Indeterminate Nonspecific Pathological	64 12.8% 171 34.2% 265 53.0%	18 13.0% 43 31.2% 77 55.8%	0.79

**Fig. 3:**
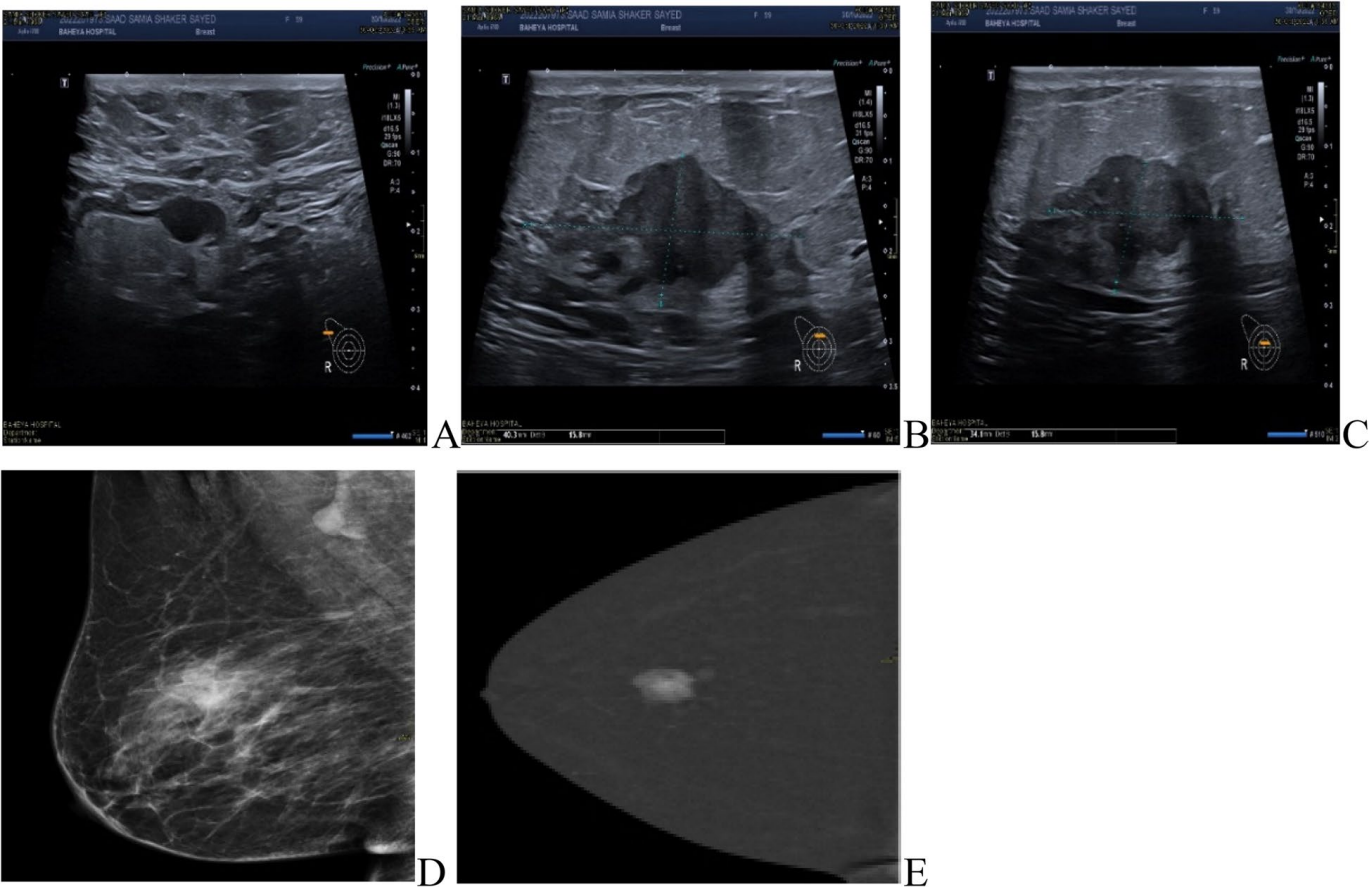
Further imaging modalities. **A**, **B**, **C** 60-years-old female patient had right breast irregular hypoechoic solid mass by ultrasound (BIRADS 5). **D**, **E** CESM showed a right breast irregular heterogeneously enhancing solid mass

**Fig. 4:**
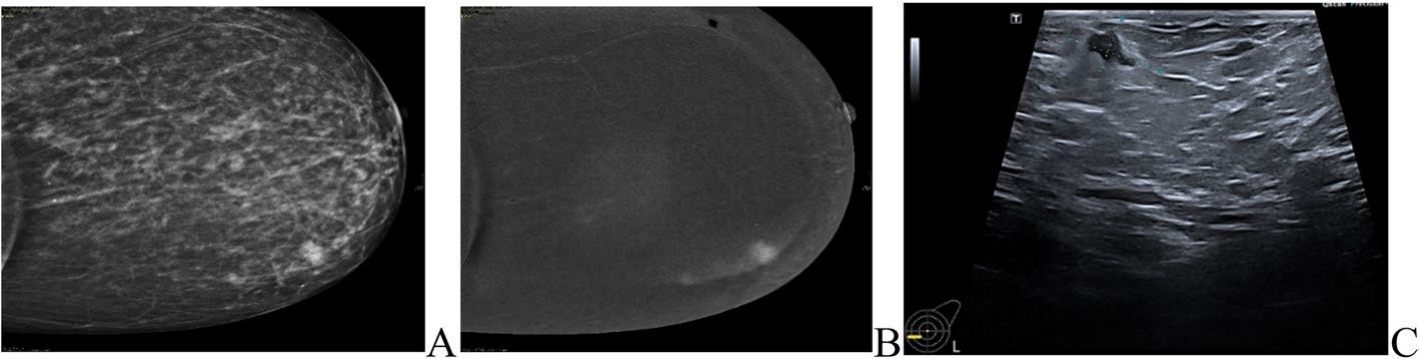
Role of CESM in diagnosis of IMPC patients. **A**, **B** 42-years-old patient presented with a left LIQ irregular spiculated mass with suspicious microcalcifications, other similar lesions were seen anterior and posterior at the same line. **C** Ultrasound showed a heterogeneously hypoechoic irregular mass with a spiculated outline with multiple similar satellite lesions were seen anterior and posterior to the main lesions

MRI was done for 5 cases (3.6%), while CESM was performed for 18 cases (13%) of the IMPC group, the commonest presentation of IMPC in contrast study was irregular shaped enhanced mass in 21 patients and non-mass enhancement was found in 5 patients. Figs. (3, 4).

The average tumor size in the IMPC and IDC groups was 3.37 ± 2.04 cm and 2.72 ± 1.39 cm, respectively (*P* < 0.001).

The percentage of tumors larger than 5cm, was reported 9.5% in IMPC and 7.4% in IDC.

The pure form of IMPC was the most common type and found in 90 cases (65%) and 47 cases (34%) were mixed type where IDC was the commonest associated type.

There are 6 cases in the IMPC group diagnosed as invasive mucinous carcinoma on biopsy, then in the specimen was mixed invasive micropapillary, IBC-NST and invasive mucinous carcinoma.

On core biopsy, 28 cases were diagnosed as IMPC with focal IDC component, but in corresponding specimens 10 cases were only approved to be mixed invasive micropapillary and invasive duct carcinoma, while others diagnosed as pure invasive micropapillary carcinoma without IDC component.

On the other hand, 48 of our cases were diagnosed as IDC on core biopsy, but in the final specimen examination, 17 of these cases were diagnosed as pure invasive micropapillary carcinoma without invasive ductal component.

The explanation of controversy in proper histologic subtyping of carcinoma on core biopsy and the definite subtype on the corresponding specimen was that the ductal component which only represented in the biopsy is a very minor component of the tumor or the limited sampling, tissue fragmentation and architecture distortion in core biopsy may cause diagnostic pitfalls as regard precise subtyping of the tumor.

The incidence of LVI in the IMPC group was 88.3% in comparison to 47.0% in the IDC group (*p* < 0.001).

IMPC had a higher incidence of lymph node involvement than the IDC group (68.8% and 56% respectively) with N3 stage reported in 12.4% of IMPC patients.

IMPC had a higher nuclear grade than the IDC group (25.1% and 15.2% respectively).

**Fig. 5:**
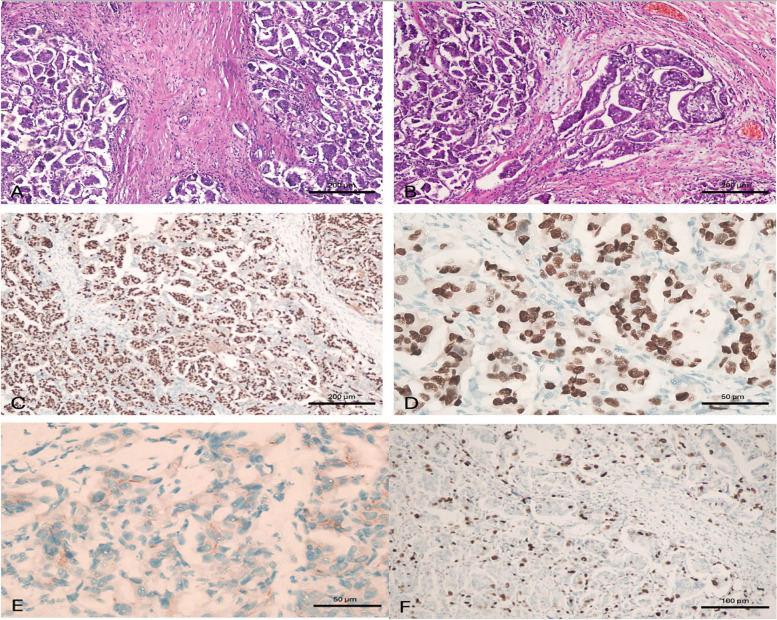
A case of invasive micropapillary carcinoma. A case of invasive micropapillary carcinoma, grade II. **A** Tissue core biopsy, × 100, **B** MRM specimen × 100 with Positive metastatic L. nodes 2/15, **C** ER is positive in > 90% of tumor cells, × 100, **D** PR is positive in > 90% of tumor cells, × 400, **E** HER2/neu is negative, × 400 and **F)** Ki-67 labelling index is high, × 200. This case was considered as luminal type pure invasive micropapillary carcinoma. (100 micron 20__ 50 micron 40)

**Fig. 6:**
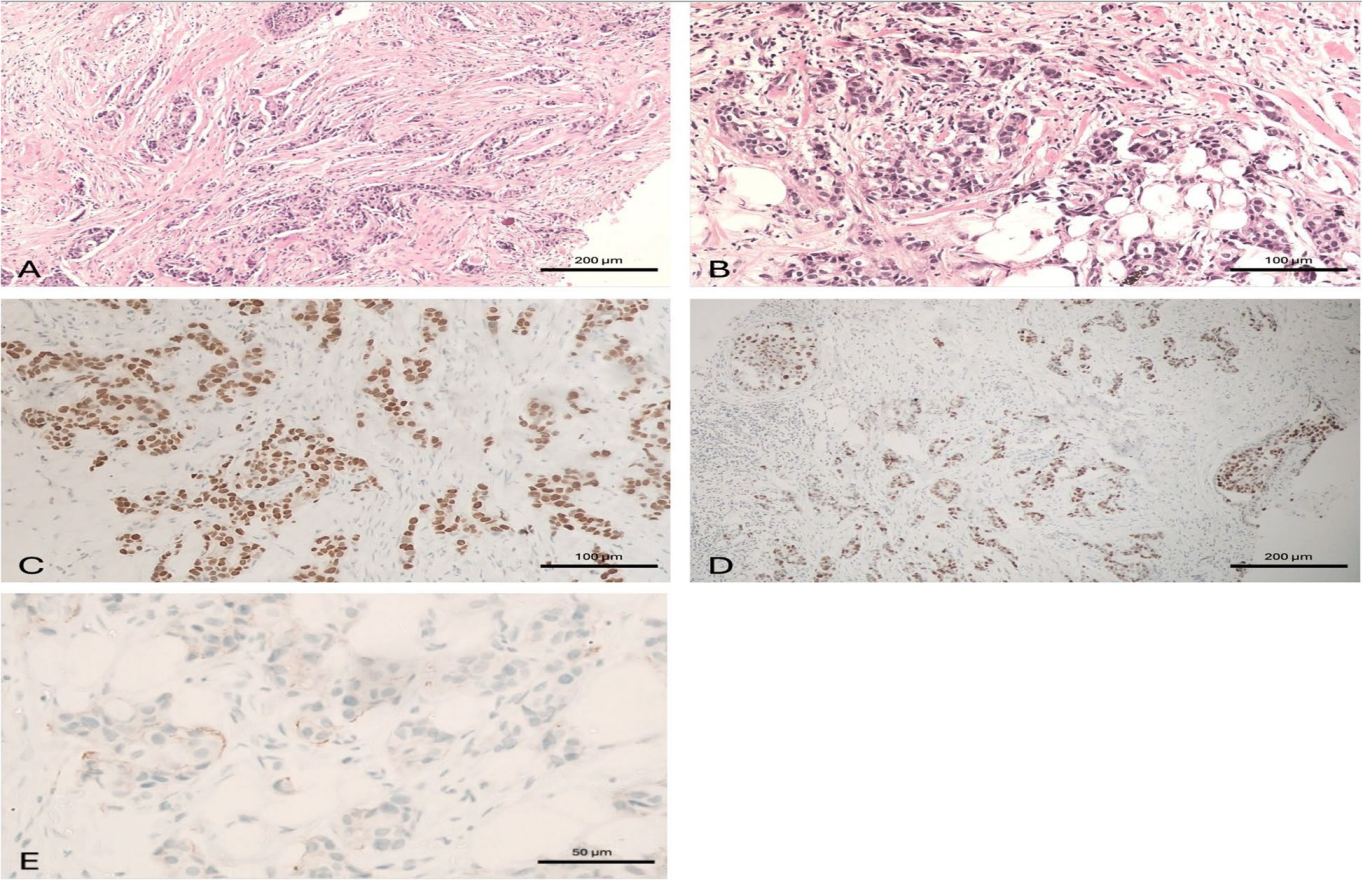
A case of invasive duct carcinoma. A case of invasive duct carcinoma, grade II. **A** Tissue core biopsy, × 100, **B** MRM specimen, × 200 with negative L. nodes 0/16, **C** ER is positive in > 90% of tumor cells, × 200, **D** PR is positive in > 90% of tumor cells, × 100, **E** HER2/neu is negative, × 400. This case was considered as luminal type pure invasive duct carcinoma

The percentage of ER-positive patients was 97.8% in the IMPC group and 87.6% in the IDC group (*p* < 0.001), while PR-positive cases were 98.6% in the IMPC group and 88.8% in the IDC group (*p* < 0.001). HER2 status was positive in 4.3% of IMPCs and 8% of IDCs (*p* = 0.23) (Table 3) (Figs. 5, 6).

**Table 3:** The pathological features of the IMPC and IDC group

	IDC *N* = 500	Micropapillary *N* = 138	*p*-value
Pathology (Single/MC/MF) MC MF Single	21 4.2% 14 2.8% 465 93.0%	14 10.1% 29 21.0% 94 68.1%	< 0.001
Tumor size /cm	2.72 ± 1.395	3.37 ± 2.046	< 0.001
Invasive tumor size	2.30 ± 1.50	2.62 ± 1.76	0.03
Grad I II III	29 5.7% 346 69.2% 125 25.1%	0 0.0% 116 84.1% 21 15.2%	< 0.001
DCIS in specimen No Yes	285 57% 215 43%	38 27.7% 99 72.3%	
Pathological T Tis Tmi T0 T1 T2 T3 T4	7 1.4% 0 0.0% 5 1.0% 244 48.8% 207 41.6% 26 5.2% 11 2.2%	0 0.0% 2 1.4% 4 2.9% 66 47.8% 52 38.0% 9 6.6% 4 2.9%	0.002
Pathological N N0 N1 N2 N3 Nx	220 44.0% 169 33.8% 68 13.7% 43 8.6% 0 0.0%	39 28.5% 43 31.4% 35 25.5% 17 12.4% 2 1.4%	0.001
ER Negative positive	62 12.4% 438 87.6%	3 2.2% 135 97.8%	< 0.001
PR Negative positive	56 11.2% 444 88.8%	2 1.4% 136 98.6%	< 0.001
HER2 Negative Positive	460 92% 40 8%	132 95.7% 6 4.3%	0.23
Lymphovascular invasion	189 47.0%	121 88.3%	< 0.001

Regarding definitive surgical management, IMPC had a lower rate of breast conserving surgery (26% vs.37.8%) compared with IDC. While, 49.3% of IMPC patients underwent modified radical mastectomy in comparison to 46% of the IDC patients. Such high incidence of mastectomy was due to the advanced stage at presentation, presence of multiple lesions and presence of intra-ductal extension with nipple involvement.

The incidence of re-surgery in the IMPC group was only in 3 cases, two of them underwent completion mastectomy after the initial conservative breast surgery and axillary clearance. While one patient underwent wider margin excision as positive margin for an invasive residual disease was found.

Two patients in the IMPC group had distant metastasis at the initial diagnosis, they had multiple metastatic lesions and received systemic treatment but one of them underwent palliative mastectomy.

Systemic chemotherapy was administered to 107 patients (77.5%) in the IMPC group and to 207 patients (41%) in the IDC group. Hormonal therapy was administered to all IMPC patients and 76% patients in the IDC group (Table 4).

**Table 4:** Surgical management and adjuvant treatment

	IDC *N* = 500	Micropapillary *N* = 138	*p*-value
Type of definitive breast surgery MRM CBS(WLE) Mastectomy with reconstruction Simple mastectomy	230 46% 189 37.8% 11 2.2% 70 14.0%	67 49.3% 36 26% 5 3.7% 29 21.3%	0.03
Axillary surgery AC SLNB	311 62.2% 189 37.8%	84 61.8% 52 38.2%	0.92
Neoadjuvant	180 36.0%	48 34.8%	0.79
Chemotherapy No Yes	293 58.6% 207 41.4%	30 21.7% 107 77.5%	< 0.001
Radiotherapy No Yes	203 40.6% 297 59.4%	30 21.7% 107 77.5%	< 0.001
Hormonal No Yes	118 23.6% 382 76.4%	0 0.0% 138 100.0%	< 0.001

The overall median follow-up duration was 21 months (range 6 – 88 months) with mean follow up duration = 29.8months.

Among the 138 IMPC patients, local recurrence developed in 3 cases, they developed a recurrence at 6,18 and 48 months postoperative. Distant metastasis developed in 5 patients in the form of bone, lung, hepatic and mediastinal lymph node metastasis.

The survival analysis indicated that IMPC patients had no significant difference in overall survival compared with IDC patients and no differences were noted in locoregional recurrence rate comparing IMPCs with IDCs (2.2% and 0.4% respectively). *P* value for local recurrence = 0.12 (yates corrected chi square).

Distant metastasis rate comparing IMPCs with IDCs was (3.7% and 5.4% respectively). *P* value for distant metastasis = 0.53 (Table 5).

**Table 5:** Survival and follow up data of the studied groups

	IDC *N* = 500		Micropapillary *N* = 138		*p*-value
No of deaths Alive Dead	429	85.8	135	97.8	0.35
	17	3.4	3	2.2	
Recurrence Local recurrence Distant metastasis	2	0.4%	3	2.2%	0.12
	27	5.4%	5	3.7%	0.53

Comparison of OS between IDC and micropapillary cases (Matched by propensity score matching -PSM).

**Table:** Case Processing Summary

Type	Total N	N of Events	Censored	
			N	Percent
IDC	125	7	118	94.4%
Micropapillary	128	3	125	97.7%
Overall	253	10	243	96.0%

**Table:** 

Type	Mean survival time^a^			
	Estimate	Std. Error	95% Confidence Interval	
			Lower Bound	Upper Bound
IDC	84.596	2.314	80.061	89.131
Micropapillary	57.530	.844	55.876	59.185
Overall	85.807	1.633	82.606	89.008

**Table:** Overall Comparisons

	Chi-Square	df	Sig.
Log Rank (Mantel-Cox)	.438	1	.508

Test of equality of survival distributions for the different levels type

Disease free survival


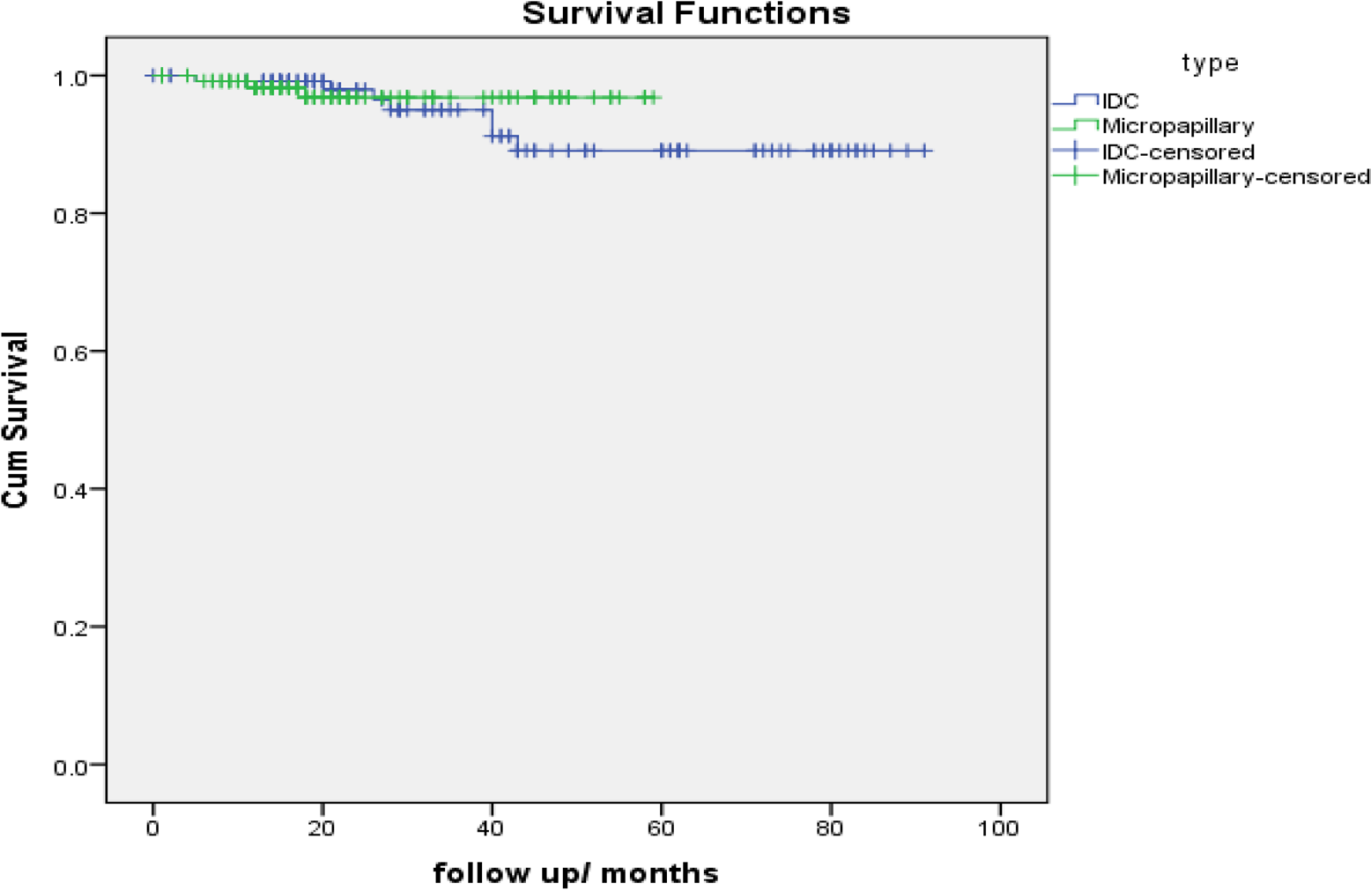

**Table:** Case Processing Summary

Type	Total N	N of Events	Censored	
			N	Percent
IDC	124	11	113	91.1%
Micropapillary	129	5	124	96.1%
Overall	253	16	237	93.7%

**Table:** 

Type	Mean^a^			
	Estimate	Std. Error	95% Confidence Interval	
			Lower Bound	Upper Bound
IDC	77.324	3.019	71.407	83.242
Micropapillary	56.062	1.355	53.407	58.718
Overall	78.725	2.333	74.152	83.299

**Table:** Overall Comparisons

	Chi-Square	df	Sig.
Log Rank (Mantel-Cox)	.380	1	.537

Test of equality of survival distributions for the different levels of type


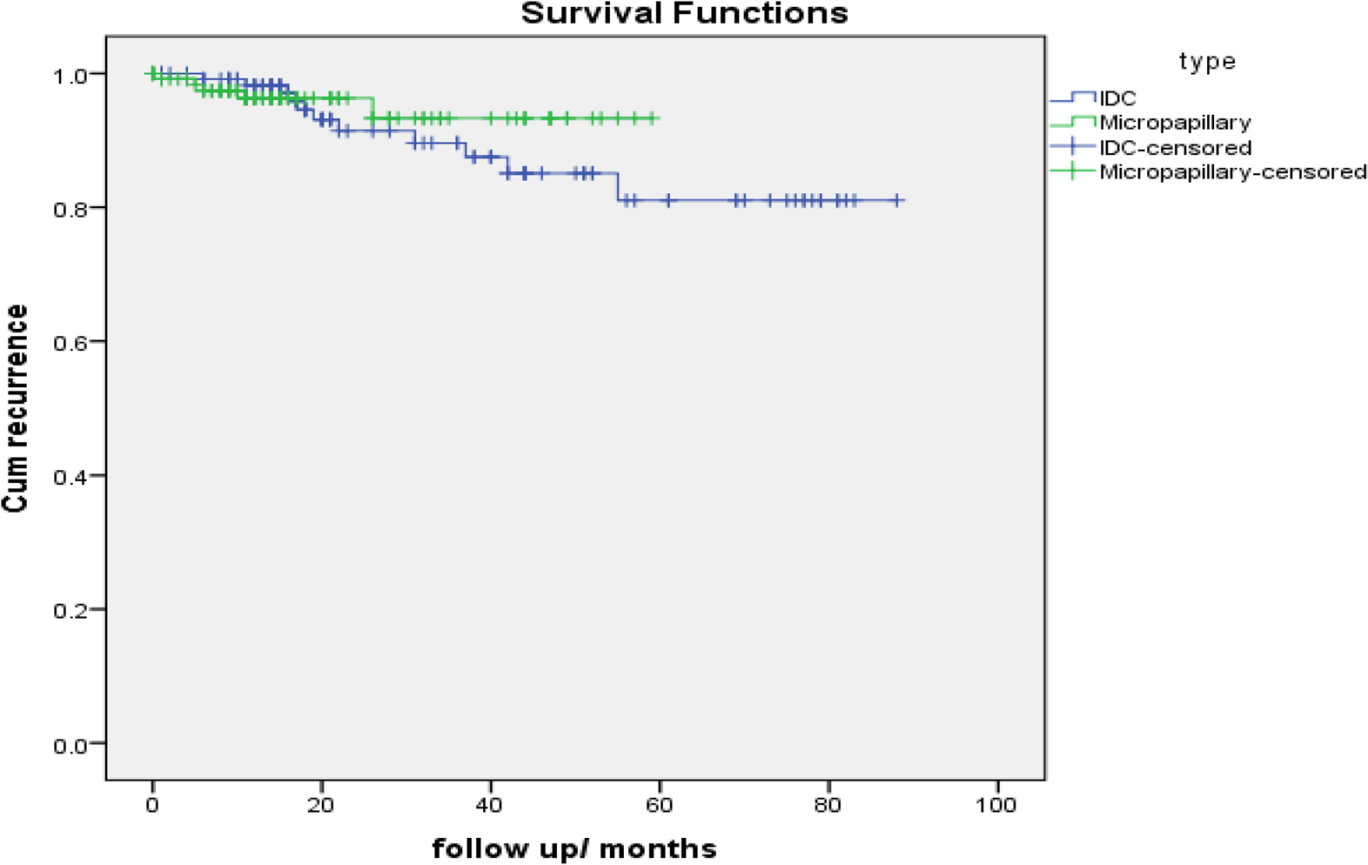


## Discussion

IMPC is a highly invasive type of breast cancer. Hashmi A.A. et al. [13] found that the incidence of IMPC is very low accounting for 0.76–3.8% of breast carcinomas.

Shi WB et al.; [7] in a study comparing 188 IMPC cases and 1,289 invasive ductal carcinoma (IDC) cases from China showed that IMPC can occur either alone or mixed with other histological types, such as ductal carcinoma in situ, mucinous carcinoma and IDC. Furthermore, the majority of patients had mixed IMPC.

Fakhry et al. [14] reported that 64.7% of IMPC patients were pure type. In our study, we found that the pure form of IMPC was the commonest type and presented in 90 patients (65%) and 47 cases (34%) were mixed type which was similar to that reported by Nassar et al. [15], and Guo et al. [16] in their studies.

In our study, the commonest finding of IMPC on breast mammography was an irregular shaped mass with a non-circumscribed spiculated margin. While, the commonest sonographic finding of IMPC was hypoechoic mass with irregular shapes and spiculated margins.

These findings were similar to the results demonstrated by Jones et al., [17] which found that the commonest morphologic finding of IMPC was an irregular high-density lesion (50% of patients) with spiculated margin (42% of patients). However, Günhan-Bilgen et al. [18] reported that an ovoid or round lesion was found in 53.8% of patients.

Alsharif et al., [19] reported that the commonest sonographic finding of IMPC was hypoechoic masse (39/41, 95%) with irregular shape (30/41, 73.2%) and angular or spiculated margin (26/41, 63.4%).

In our study, MRI was done for 5 cases (3.6%), while CESM was performed for 18 cases (13%) of the IMPC group, the commonest presentation of IMPC in contrast study was irregular shaped enhanced lesion in 21 cases and non-mass enhancement was presented in 5 cases.

Nangogn et al. [20] and yoon et al. [8] recorded that the commonest finding of IMPCs in MRI was spiculated irregular mass with early rapid initial heterogenous enhancement, indicating that the MRI findings correlated with the invasiveness of IMPC.

Fakhry et al. [14] conducted a study on 68 cases, out of which 17 cases underwent CEM. In all of these cases, the masses showed pathological enhancement, which was either in the form of mass enhancement (12/17 patients, 70.6%) or non-mass enhancement (4/17 patients, 23.5%). The majority of the enhanced masses were irregular in shape (11/12 patients, 91.7%).

All patients underwent axillary sonography and 77 patients (55.8%) of the IMPC group exhibited pathological lymph nodes; this percentage was similar to that recorded by Nangong et al. [20] which was 54.8% and lower than that recorded by Jones et al. [17] but higher than that of Günhan et al. [18] which were 67% and 38% respectively.

Günhan et al. [18] reported microcalcification in about 66.7% of the cases. In our study, associated microcalcifications were found in 49 patients (35.5%) of the IMPC group. Yun et al. [21] and Adrada et al. [22] showed a fine pleomorphic appearance (66.7% and 68%).

Hao et al. [23] compared the rate of tumors larger than 5cm, reporting 3% in IDC and 4.3% in IMPC. In our study, the rate of tumors larger than 5cm, was reported 7.4% in the IDC patients and 9.5% in the IMPC patients.

Yu et al., et al. [24] documented in a study comparing 72 cases of IMPC and 144 cases of IDC of the breast that IMPC had a higher nuclear grade than IDC (52.8% vs. 37.5% respectively). In our study, IMPC had a higher nuclear grade than the IDC group (25.1% and 15.2% respectively).

Verras GI et al.; [9] demonstrated that IMPC was an aggressive breast cancer subtype with a great tendency to lymphovascular invasion and lymph node metastasis. In our study, the incidence of LVI in the IMPC patients was 88.3% in comparison to 47.0% in the IDC patients (*p* < 0.001). Tang et al., [25] also reported that lymphovascular involvement was more common among the IIMPC group than IDC group, with a percentage of 14.7% compared to only 0.1% in the IDC group.

Also, Shi et al. [7] reported that LVI was detected in 74.5% of cases. Furthermore, the frequency of LVI was found to be greater in IMPC cases when compared to IDC cases. Jones et al., [17] recorded angiolymphatic invasion in 69% of cases.

Hashmi et al. [13] reported in his comparative study that nodal involvement was present in 49.5% of IDC patients and N3 stage was only 15.6% in IDC patients compared to 33% in IMPC patients. In our study, the percentage of lymph node involvement of IMPC and IDC patients were 68.8% and 56% respectively with N3 stage reported in 12.4% of IMPC patients.

Guan et al. [26], Lewis et al., [27], Pettinato et al., [28] and De La Cruz et al., [29] recorded a higher percentage of lymph node metastasis in IMPC patients, reaching 90%, 92.9%,55.2% and 60.9% respectively.

The management of IMPC remains controversial, particularly among breast surgeons. Modified radical mastectomy was the preferred surgical procedure for the majority of IMPC case reports, as found in a study conducted by Yu et al., [24] where 99% of IMPC cases underwent modified radical mastectomy. Fakhry et al. [14] reported that 76.5% of the patients underwent modified radical mastectomy. In our study, 49.3% of IMPC patients received modified radical mastectomy.

IMPC patients were also prone to accept BCS rather than mastectomy in the previous series conducted by Lewis GD,et al. [27] and Vingiani, A. et al. [30]. However, the precise prognosis value of BCS for patients with IMPC remained unknowable. In our study, IMPC had a lower rate of breast conserving surgery (26% vs.37.8%) compared with IDC.

IMPC was characterized by a high incidence of ER and PR positivity. Our study recorded a high percentage of ER (97.8%) and PR (98.6%) expression. Our findings are similar to those found by Walsh et al., [31] who reported ER and PR expression of 90% and 70%, respectively. Zekioglu et al. [32] demonstrated a rate of ER and PR expression of 68% and 61%respectively.

In this study, we reported a relatively lower percentage of HER-2 positivity (4.3%). Also, Nangong et al. [20] showed HER 2 overexpression in 26.4% of cases.

However, Cui et al. [33] reported a much higher incidence of HER 2 positivity and Perron et al., [34] reported that 65% of IMPCs were HER-2 positive.

Chen, A et al. [35] reported that that the percentage of radiation therapy for IMPC patients was similar to those seen in IDC patients and demonstrates a similar benefit of radiation treatment in both groups. In our study,77.5% patients received radiotherapy in IMPC group in compared to 59.4% patients in IDC group.

Shi et al. [7] found that patients with IMPC had worse recurrence-free survival (RFS) and overall survival (OS) rates as compared to those with IDC. However, because IMPC is relatively rare, most studies had reported on small sample sizes with limited follow-ups.

Yu et al., [24] conducted a comparison between IMPC and IDC patients, and the results showed that the IMPC group had a greater tendency for LRR compared to the IDC group (*P* = 0.03), but the distant metastasis rate (*P* = 0.52) and OS rate (*P* = 0.67) of the IMPC showed no statistical differences from the IDC group.

Nevertheless, several recent studies documented that IMPC had better or similar prognosis in comparison to IDC.

Hao et al. [23] and Vingiani et al. [30] documented that there was no statistically significant difference in OS and disease-free survival between IMPC patients and IDC patients which was similar to our results. locoregional recurrence rate comparing IMPCs with IDCs was (2.2% and 0.4% respectively). *P* value for local recurrence = 0.12 (yates corrected chi square). Distant metastasis rate comparing IMPCs with IDCs was (3.7% and 5.4% respectively). *P* value for distant metastasis = 0.53.

Chen H et al. [36], compared the overall survival in patient groups with similar nodal involvement and found that IMPC group had better breast cancer–specific survival and overall survival than IDC group.

## Conclusion

The results from our PSM analysis suggested that there was no statistically significant difference in prognosis between IMPC and IDC patients after matching them with similar clinical characteristics. However, IMPC was found to be more aggressive, had larger tumor size, greater lymph node metastasis rate and an advanced tumor stage.

## Data Availability

No datasets were generated or analysed during the current study.

## Abbreviations

IMPC: Invasive micropapillary carcinoma
IDC: Invasive duct carcinoma
MRM: Modified radical mastectomy
CBS: Conserving breast surgery
ER: Estrogen receptor
PR: Progesterone receptor
LVI: Lymphovascular invasion
LN: Lymph node
CESM: Contrast enhanced spectral mammography
OS: Overall survival
DFS: Disease free survival
